# Shedding new light on early sex determination in zebrafish

**DOI:** 10.1007/s00204-020-02915-y

**Published:** 2020-09-25

**Authors:** Alex C. King, Michelle Gut, Armin K. Zenker

**Affiliations:** FHNW, University of Applied Sciences and Arts North-Western Switzerland, School of Life Sciences, Institute for Ecopreneurship, Hofackerstrasse 30, 4132 Muttenz, Switzerland

**Keywords:** Sex determination, Genes, Zebrafish, Juvenile expression

## Abstract

In contrast to established zebrafish gene annotations, the question of sex determination has still not been conclusively clarified for developing zebrafish, *Danio rerio,* larvae, 28 dpf or earlier. Recent studies indicate polygenic sex determination (PSD), with the genes being distributed throughout the genome. Early genetic markers of sex in zebrafish help unravel co-founding sex-related differences to apply to human health and environmental toxicity studies. A qPCR-based method was developed for six genes: cytochrome P450, family 17, subfamily A, polypeptide 1 (cyp17a1); cytochrome P450, family 19, subfamily A, polypeptide 1a (cyp19a1a); cytochrome P450, family 19, subfamily A, polypeptides 1b (cyp19a1b); vitellogenin 1 (vtg1); nuclear receptor subfamily 0, group B, member 1 (nr0b1), sry (sex-determining region Y)-box 9b (sox9b) and actin, beta 1 (actb1), the reference gene. Sry-box 9a (Sox9a), insulin-like growth factor 3 (igf3) and double sex and mab-3 related transcription factor 1 (dmrt1), which are also known to be associated with sex determination, were used in gene expression tests. Additionally, Next-Generation-Sequencing (NGS) sequenced the genome of two adult female and male and two juveniles. PCR analysis of adult zebrafish revealed sex-specific expression of cyp17a1, cyp19a1a, vtg1, igf3 and dmrt1, the first four strongly expressed in female zebrafish and the last one highly expressed in male conspecifics. From NGS, nine female and four male-fated genes were selected as novel for assessing zebrafish sex, 28 dpf. Differences in transcriptomes allowed allocation of sex-specific genes also expressed in juvenile zebrafish.

**Electronic supplementary material:**

The online version of this article (10.1007/s00204-020-02915-y) contains supplementary material, which is available to authorized users.

## Introduction

Fish embryos are an attractive model for the risk assessment of chemicals and for the investigation of effects of endocrine disrupting substances (Kazeto et al. 2004; Yu et al. 2018) and drug discovery (Vaz et al. 2018). Toxicological studies which test acute toxicity by means of Fish Embryo Acute Test (FET) (OECD 2013) or Early-life Stage Toxicity Tests (ELS Test) (OECD 2010) often use zebrafish embryos as indicators of endpoints (Kazeto et al. 2004; Yu et al. 2018). At this early stage, the embryos cannot be morphologically sexed because during the test period sex differentiation is not yet complete (Kimmel et al. 1995). Even in zebrafish larvae, 96 h post fertilisation (hpf), many genes are insufficiently expressed, making genetic sex determination difficult. However, the sex of the individual embryo could likely be a confounding factor masking the underlying impact of a test. The make-up of the individuals in a chosen population is random and contains an unknown mix of both males and females. Consideration of this mixture could therefore be important, as studies have shown gender-specific differences in zebrafish and if not taken into consideration tests could have misleading results (Kling et al. 2008; Dlugos et al. 2011). Only tests such as the 21-day fish assay (OECD 2009), which use sexually mature zebrafish, can take these gender-specific effects into account because the zebrafish are at a stage at which the sex can be determined visually. ELS tests are a replacement for adult fish tests because of animal welfare issues (Nagel 2002) so it is increasingly import to identify early gene markers of sex. Therefore, establishment of early sex-determining genes in zebrafish is vital to ensure that discrepancies in the resulting impact can be distinguished, so that confounding factors are revealed and the issue of sex-related difference is evident.

It is known from human drug development and use of medication that there are differences in sensitivity between male and females (Soldin and Mattison 2009; Whitley and Lindsey 2009; Parekh et al. 2011; NIDA National Institute on Drug Abuse 2017). There are also varying effects of trials on zebrafish of different sex (Brian et al. 2005; Tilton et al. 2008; Balik-Meisner et al. 2018) which makes it difficult to interpret data if you use the early life stage zebrafish model for drug discovery and toxicity testing for general health and environmental benefits. There are few studies, which have revealed early identifiable genes for sex determination in zebrafish (Liew 2013; Kossack and Draper 2016). This highlights the need for identification of novel genes for sex identification at early stages in zebrafish to scrutinise sex-specific drug susceptibility and translate to human data.

The early gonadal development of zebrafish is a complex process (see Table 1 S1, supporting information). The zebrafish is termed a juvenile hermaphrodite; all gonads initially develop as an undifferentiated juvenile ovary, which are ovary-like organs with oocyte-like germ cells that later degrade and transform into true ovary or testis (Takahashi 1976; Uchida et al. 2002; Maack and Segner 2003; Wang et al. 2007; Yang et al. 2017). During the female‐to‐male differentiation, intersex gonads contain both oocyte‐like germ cells and developing testicular tissues; oocytes regress by apoptosis from 19–27 dpf (Uchida et al. 2002; Chen and Ge 2013; Wilson et al. 2014). Development of spermatogonia and proliferation of stromal somatic cells in the transforming gonad lead to final differentiation into the testis and the fish becomes male (Uchida et al. 2002; Chen and Ge 2013). Remarkably, after oocyte depletion, early oocyte-producing individuals and those who reproduce as females can also change into fertile males, indicating the need for oocytes, not only for initial sex determination but also for maintenance of the adult female zebrafish phenotype (Dranow et al. 2016).

The first indication of zebrafish sex differentiation is at 10–12 dpf when the ovarian gonocyte proliferates and differentiates. At 10–17 dpf, somatic genes express indifferently, becoming sexually dimorphic at three weeks (Tong et al. 2010). Zebrafish gonad differentiation begins around 25 dpf (Uchida et al. 2002; Wang et al. 2007; Chen and Ge 2013) but it has also been reported at a much earlier stage in development, 14 dpf (Hsiao and Tsai 2003). Gonads can be morphologically identified and differentiation of the gonad is complete at around 35 dpf in females and 45 dpf in males (Uchida et al. 2002; Wang et al. 2007; Chen and Ge 2013; Wong et al. 2014). The variation among studies regarding age at which sex-determining genes are established indicates differing factors which may significantly influence the timing of sex differentiation in the zebrafish (Chen and Ge 2013). In teleost fish, gonadal sex differentiation is highly plastic and can be influenced by both genetic and environmental factors or a combination of both (Devlin and Nagahama 2002; Strüssmann and Nakamura 2003; Chen et al. 2017; Guiguen et al. 2019; Valdivieso et al. 2019). Despite morphological and histological zebrafish gonadal differentiation being well documented (Takahashi 1976; Uchida et al. 2002; Wang et al. 2007), the molecular mechanism of sex determination remains largely unknown (Tong et al. 2010; Chen et al. 2017).

Genetic factors are key in influencing the sexual fate of zebrafish. There has been relatively little discovery of discernible sex chromosomes in zebrafish and distinguishing the sex of juveniles is challenging (Liew et al. 2012; Liew 2013). Zebrafish have neither heteromorphic sex chromosomes, which are common in mammals (Traut and Winking 2001), nor a single specific sex-determining locus (Liew et al. 2012). The entire genome has been sequenced and more than 26,000 annotated genes are known (Zeng and Gong 2002; Li et al. 2004; Wen et al. 2005; Knoll-Gellida et al. 2006; Santos et al. 2007; Jørgensen et al. 2008; Sreenivasan et al. 2008; Groh et al. 2011; Collins et al. 2012; Howe et al. 2013), but questions remain about the complicated nature of sex-determination in zebrafish. Recent studies have pointed to PSD in zebrafish in which multiple genes along with the influences of primordial germ cells dictate the sexual fate of zebrafish (Von Hofsten and Olsson 2005; Anderson et al. 2012; Liew et al. 2012; Liew 2013; Nagabhushana and Mishra 2016; Chen et al. 2017; Yang et al. 2017). Genes contributing to sex determination and gonadal differentiation are distributed throughout the genome, with the combination and interaction of this network of alleles establishing the sex of the individual (Bulmer and Bull 1982; Liew et al. 2012; Wilson et al. 2014; Crowder et al. 2018b).

In many pathways genetic expression is changed with age and expressed differently in males and females (Arslan-Ergul and Adams 2014). Female-dominant genetic factors are necessary for zebrafish sex determination (Tong et al. 2010). Several genes, including aromatase, *cyp19a1a* and forkhead box L2a (f*oxl2a)* promote ovary differentiation and development (Siegfried and Nüsslein-Volhard 2008; Clelland and Peng 2009; Dranow et al. 2016; Chen et al. 2017). In addition, sex differentiation can be biased in favour of fully functioning and fertile females when juvenile zebrafish are exposed to exogenous oestrogens (Örn et al. 2006; Schulz et al. 2007; Crowder et al. 2018b). On the other hand, male sex determination is initiated by expression of sex-determining genes that activate downstream factors essential for testis development and spermatogenesis, anti-Mullerian hormones (*amh*);* dmrt1* and sry-related HMGbox 9 (*sox9*) (Rodríguez-Marí et al. 2005; Schulz et al. 2007; Herpin and Schartl 2015; Jie and Jian-fang 2015; Lin et al. 2017a).

As well as genetic elements, environmental perturbations contribute to sex-fate in zebrafish. In fish, development of the gonad may be influenced by fluctuations in intrinsic factors such as growth or behaviour, or by extrinsic environmental factors (Devlin and Nagahama 2002). Sex ratio and sex determination in zebrafish populations is known to be altered by both abiotic and biotic environmental factors (Valdivieso et al. 2019). Stress is caused by: changes in water temperature (Uchida et al. 2004; Hörstgen-schwark 2011; Hörstgen-schwark et al. 2012; Brown et al. 2015; Ribas et al. 2016; Santos et al. 2017) and pH; hypoxia (Shang and Wu 2004; Shang et al. 2006; Lo et al. 2011; Robertson et al. 2014); endocrine disrupting chemicals (EDCs) including endocrine disrupting hormones (EDHs) xenoestrogens and 17a-ethinylestradiol; aromatase inhibitors fadrozole and xenoandrogens; 17b-trenbolone; pollution; high densities (Hazlerigg et al. 2012; Liew et al. 2012; Ribas et al. 2017) and reduction in food (Lawrence et al. 2008). In turn, stress is associated with zebrafish masculinisation, but these results are conflicting (Ribas et al. 2017; Santos et al. 2017; Crowder et al. 2018a).

Environmental stress during zebrafish embryogenesis can produce a male-biased sex ratio (Uchida et al. 2004; Hörstgen-schwark et al. 2012; Brown et al. 2015; Ribas et al. 2017). Gonadal masculinisation in laboratory zebrafish is visible in transcriptome data of gonads exposed to increased water temperatures, with an upregulation of male genes and repression of female-related genes. This evidence shows that sex is not only influenced by, but can be altered by, environmental factors. It also supports the theory of PSD working together with environmental factors to influence sex-fate in zebrafish (Ribas et al. 2016; Hosseini et al. 2019; Valdivieso 2019).

During recent decades, domestication of zebrafish and selection by researchers has caused alteration in natural genetic and environmental cues and consequently led to either evolution of new sex-determining methods or changes in underlying genetic sex-determining mechanisms (Guryev et al. 2006; Whiteley et al. 2011; Wilson et al. 2014; Van Den Bos et al. 2017; Holden and Brown 2018). Observations in studies suggest that domesticated zebrafish populations consist of individuals with recessive or over-dominant male-determining alleles compared to wild populations (Delomas and Dabrowski 2018).

Domesticated laboratory strains of zebrafish lack a sex chromosome, and few sex-determining genes have been identified (Crowder et al. 2018b). Genetic studies of sex in different zebrafish strains have identified multi-loci, which are considered sex-determining regions, including a single sex-linked locus on chromosome 4 (Chr4) which was highlighted in an AB laboratory strain (Sola and Gornung 2001; Bradley et al. 2011; Anderson et al. 2012; Howe et al. 2013; Yang et al. 2017). However, loss of certain naturally-occurring genes, including Chr4, through selection has been reported in some domesticated strains, AB and TU (Wilson et al. 2014).

In this study, a qPCR was developed to analyse the expression of nine selected genes, which have been previously associated with sex determination and expressed early in embryonic development and these were compared against a reference gene. It is assumed that sex in zebrafish is poly-genetically determined and that varying levels of gene expression influence development of sexual characteristics. Accordingly, expression profiles of both adult female and male, as well as 28 day-old zebrafish were established and compared to identify sex- and age-specific gene expression. Additionally, we used NGS data of whole body tissue analysis from domesticated AB strain, adult males and females, 540 dpf, and juveniles, 28 dpf, to establish a catalogue of transcriptome expression. We used this to identify sex-determining genes and indicate those expressed as early as 28 dpf in zebrafish for applications for juvenile sex identification. This information will allow cofounding sex-related variables to be revealed for human benefits.

## Methods

### Materials

#### Juvenile and adult zebrafish

Commercially available 28 dpf and adult female and male zebrafish of the strain AB, bred by the company IES, Witterswil, were used for the establishment of the expression analysis. Fish were euthanised with 2-phenoxyethanol and preserved in ethanol until subsequent RNA isolation.

### Methods

#### Development of the TaqMan qPCR for gene expression analysis

##### Gene selection

Gender-specific genes, which are thought to contribute to sex-determination in zebrafish, were selected from previous literature. It was ensured that selected genes were expressed in the juvenile stage of zebrafish according to the zebrafish information network (Zfin.org). Based on the gene ontology and the findings from literature, nine genes were selected for analysis against the reference housekeeping gene, actb1 (see Table 2 S2, supporting information).

##### Primer and TaqMan probe development

For the genes actb1, cyp17a1, nr0b1, sox9b, vtg1, cyp19a1b and cyp19a1a novel primers and probes were developed based on the NCBI reference sequences (see Table 2 S2, supporting information). These were tested for efficiency and specificity and optimised. For the genes dmrt1, sox9a and igf3, commercially available TaqMan qPCR mix (Thermo Fisher Scientific, Switzerland) was used.

##### RNA isolation—RNeasy method

Total RNA isolation from adult fish and juvenile fish, 28 dpf, was performed using Qiagen RNeasy Maxi Kit and Mini Kit, respectively, according to the manufacturer's protocol. The RNA was eluted in nuclease free water and then stored at − 80 °C. After isolation, the RNA quality was analysed by measuring the RNA concentration and determining RNA integrity by gel electrophoresis before further use. Total RNA isolated from zebrafish, which had been preserved and stored, was intact and not degraded after storage so it could be used in further tests.

##### cDNA synthesis

The mRNA was transcribed into cDNA using reverse transcriptase (RT) according to the Promega M-MLV RT protocol and oligo-dT primers. In contrast to the protocol, no nuclease inhibitor was added to the reaction; 500 ng of RNA was used instead of 2 μg RNA and a dNTP mix with a concentration of 2 mM each, total 8 mM was used, instead of single dNTPs. The synthesised cDNA was diluted in a ratio of 1:3 with nuclease free water and stored at − 20 °C before further use. The cDNA synthesis of tested juvenile and adult zebrafish RNA which was isolated with the RNeasy method and integrated DNase I digestion was used in further tests. Instead of carrying out the RT control for each individual sample, an RNA pool of a test condition of the gene expression analysis with +/− RT for each tested gene was performed.

##### TaqMan qPCR for analysis of gene expression

Sex-specific expression profiles were determined for 28 dpf and adult zebrafish, using the developed gene expression assay. A relative quantification was performed in comparison to the negative control to check whether age had an influence on the investigated genes. For each developed and commercially available probe, a master mix was prepared containing relevant primers and TaqMan probes. The qPCR analysis was performed in technical duplicates, 3 µL diluted cDNA per 15 µL qPCR reaction, according to the optimal qPCR programme. Only samples in which both replicates yielded a *C*_t_ value above the threshold of 0.0141 were used for evaluation. The mean *C*_t_ value of the replicates was used to calculate the gene expression and normalized to the reference gene, actb1.

##### RNA sequencing using NGS

To identify possible genes that contribute to sex determination, the transcriptome of adult male and adult female zebrafish were compared. Two adult male and two adult female zebrafish were selected and whole body tissue used for RNA sequencing by NGS. In addition, the RNA of two 28-day-old zebrafish was tested to gather whether they could, at this stage, be assigned to a female or male expression profile. RNA quality testing, library preparation and subsequent NGS were performed at the Biocenter under the supervision of Philippe Demougin (Life Sciences Training Facility (LSTF), Basel), according to Illumina's standard protocol for RNA sequencing (Illumina Inc., San Diego, USA, Cat. # RS-100-0801).

Comparative analysis was carried out on the NGS transcriptome data using Galaxy 77, with the latest zebrafish genome, version 35.6 downloaded from NCBI (https://www.ncbi.nlm.nih.gov/grc), used for reference against. Trimmomatic is a quality-based trimming tool, which was used to increase mapping efficiency by trimming low-quality reads from the profile. The Stringtie command assembles transcriptomes for each of the six individual files, the two 28-day juveniles, adult females and males; it defines which genes or reads transcriptomes belong to and counts them. A table of expression for all the reads was produced, showing the number of transcriptomes for each gene for each individual. Around 4 million transcriptomes per sample were generated and 24 million reads were compared across the six samples. Genes with the highest average transcriptome count between males and females were highlighted for further analysis. Integrated genomic viewer, IGV, was used to compare and visualise the expression of mapped genes for the six samples in comparison to the reference genome. The software R and the R package ggbio were used for the production of heat maps and visualised expression graphs.

## Results

### Development of qPCR for gene expression analysis—primer development, primer test and PCR optimisation

Primers and probes for seven genes; actb1, sox9b, vtg1, cyp19a1a, cyp19a1b, nr0b1 and cyp17a1 were developed, tested and optimised (see Table 3 S3, supporting information). qPCR was performed in in Rotor-Gene Q from Qiagen in three stages; 95 °C for 600 s, followed by 45 cycles at both 95 °C for 15 s and 60 °C for 60 s. Cycle threshold (*C*_t_) values obtained from the dilution series were used to determine PCR efficiency of the seven tested genes so comparisons of gene expression could be made. The developed qPCR probes, as well as commercially available probes for the genes sox9a, dmrt1 and igf3 (Thermo Fisher Scientific, Switzerland) were used for the analysis of sex-specific gene expression in zebrafish in relation to age, with actb1 used as a housekeeping reference gene.

### Gene expression analysis 28 dpf in comparison to adult male and female zebrafish

**Fig. 1:**
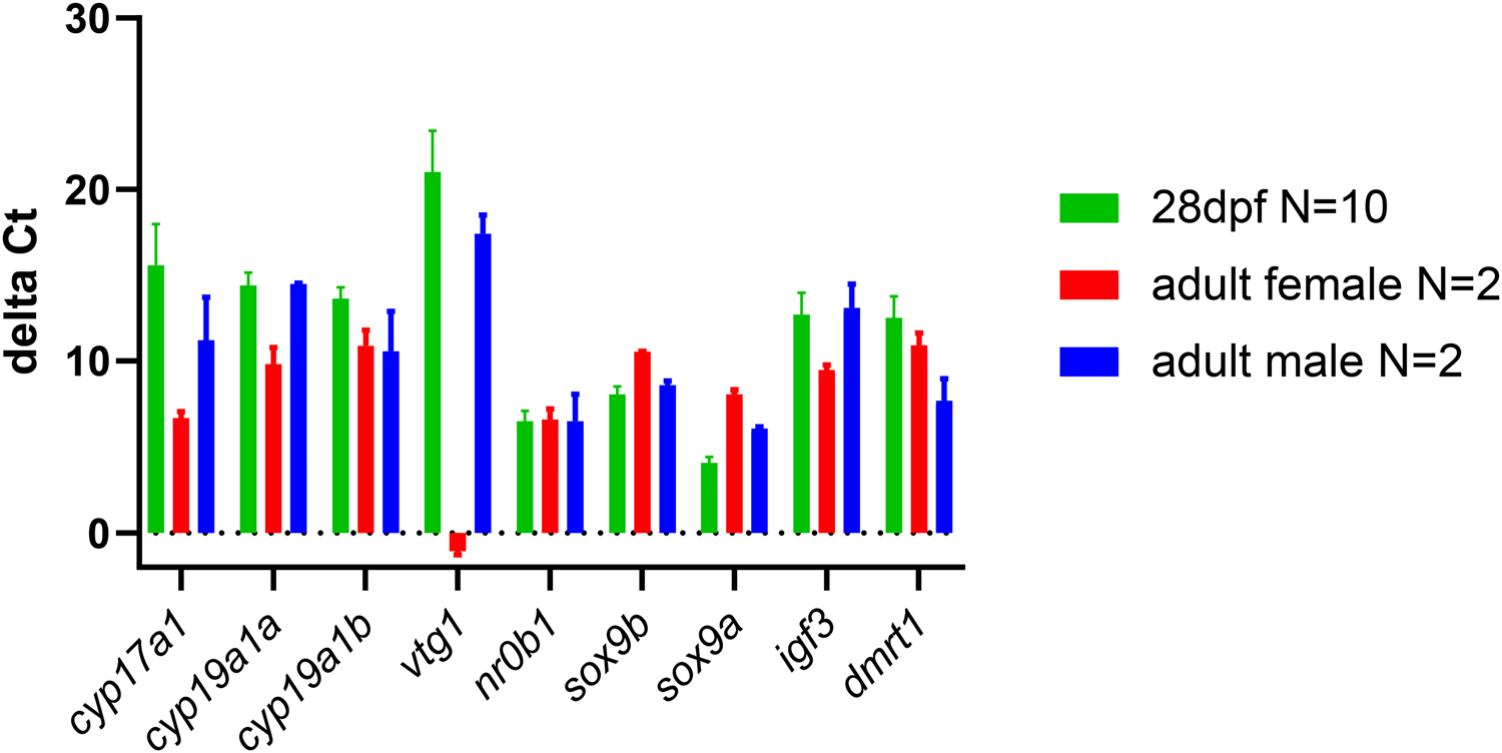
*C*_t_ value of different aged juvenile zebrafish in comparison to adult male and female zebrafish shown as a bar chart. A comparably low ∆*C*_t_ value corresponds to a strong expression and a high ∆*C*_t_ value to a weaker expression of the analysed gene

Significant differences in expression of the genes associated with sex determination were found in developing zebrafish, 28 dpf, in comparison to adult zebrafish (Fig. 1). Most strikingly, the gene expression at 28 dpf compared to sex showed a relatively low expression of vtg1 in both adult male and juvenile zebrafish, compared to females; this is also the case for cyp17a1 and igf. Additionally, the expression trend of sox9a was similar at 28 dpf and in males, with a lower expression highlighted in females (Fig. 1). Gene expression analysis of adult zebrafish is provided in S4 Figure 6, supporting information.

### RNA sequencing using NGS

**Fig. 2:**
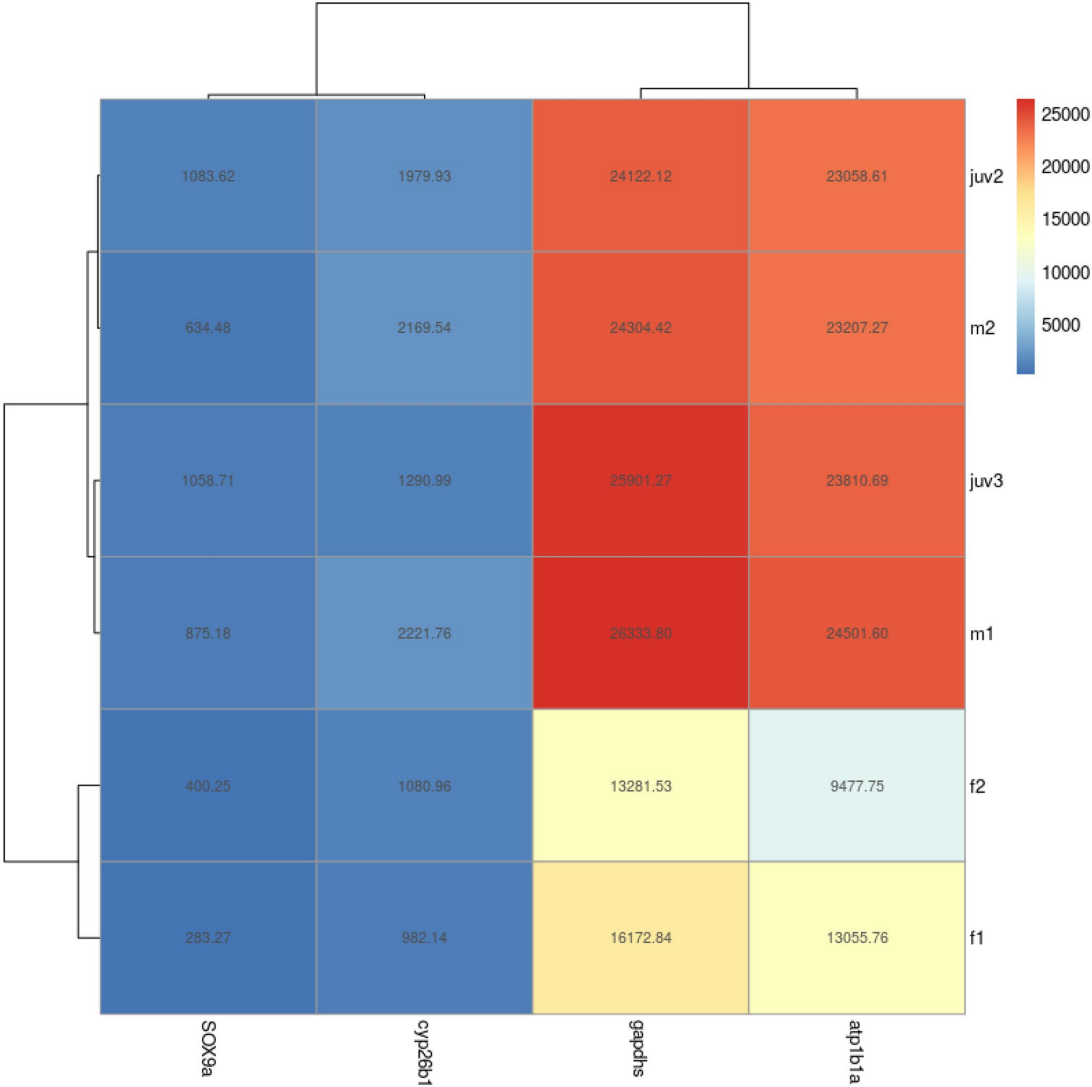
Heat map showing the relative expression of four male genes selected for early sex-determination

**Fig. 3:**
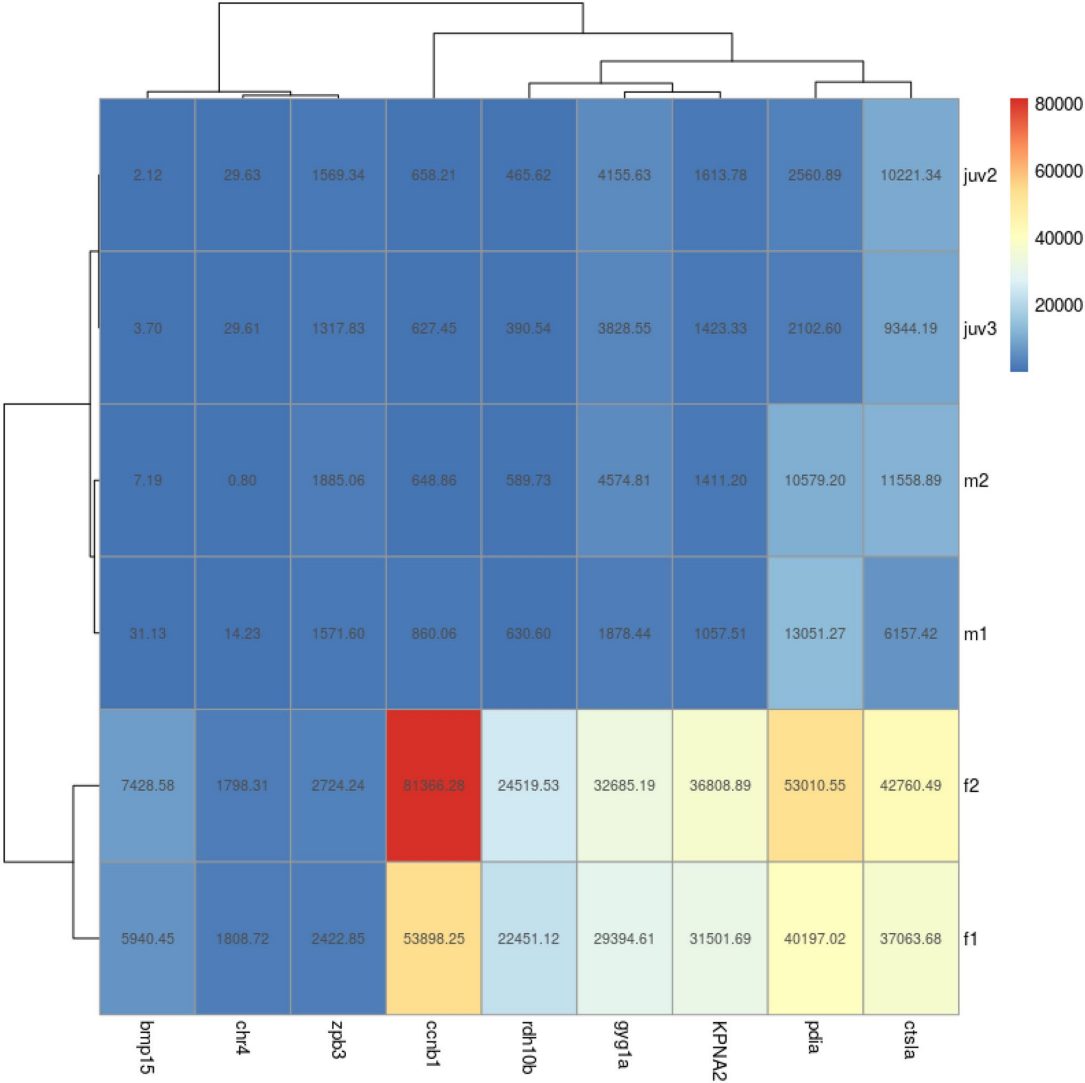
Heat map showing the relative expression of nine female genes selected for early sex-determination

RNA sequenced NGS data was evaluated for investigation of differences between the transcriptome of adult female and male zebrafish. The transcriptome dataset from the whole genome was narrowed to highlight the genes with the largest difference in transcriptome number between adult female and male zebrafish, which indicated potential sex-determining genes (see Table 4 S5, supporting information). Genes from this as well as from the PCR in this study and additional genes selected from the NGS data or known from previous research, which showed the best potential for early sex identification in zebrafish, 28 dpf, were selected. We highlight four male and nine female genes, which could be used for early sex-determination in zebrafish (Figs. 2 and 3, respectively; See also supporting information, S6 Tables 5 and 6, respectively). Further, seven female and five male additional genes were selected for their potential sex-determining role (see Table 7 S7, supporting information)**.**

### Genes selected for early female sex-determination

Six genes from high differentiation between male and females in NGS transcriptome data and three additional genes discovered from NGS data, which all are supported by previous literature, were selected as potential markers of female sex-determination in zebrafish, 28 dpf.

#### Glycogenin 1a (gyg1a)

Gyg1a was chosen for further analysis in this study as it was indicated as one of the top 50 genes with the highest expression difference in transcriptome data between males and females. Transcriptome analysis revealed that gyg1a was expressed 26 times more in female zebrafish compared with males; with juvenile expression numerically comparable to male transcriptome data (Fig. 3). Gyg1a has been linked to glycogen starch synthase activity and is involved in the glycogen biosynthetic process where it is localized to the cytoplasm (Zfin.org—ZFIN ID: ZDB-GENE-040426-2910).

#### Retinol dehydrogenase 10b (rdh10b)

The transcriptome number data described in this study shows a 39-fold increase in rdh10b in females compared with males (Fig. 3). Similarly, the average juvenile transcriptome number for the gene rdh10b is 55-fold lower than female zebrafish. Rdh10b is involved in NADP-retinol dehydrogenase and oxidoreductase activity, retinoic acid (RA) biosynthetic and retinol metabolic processes and is localized to the lipid droplet. It is expressed in several structures, including axis; notochord; pleuroperitoneal region; tail bud; and yolk syncytial layer (Zfin.org—ZFIN ID: ZDB-GENE-030909-7).

#### Protein disulphide-isomerase A4 (pdia)

For the gene, pdia, transcriptome data of female zebrafish is on average four-fold and three and a half times higher than that of male and juvenile zebrafish, respectively (Fig. 3). Pdia catalyses the re-arrangement of disulphide-isomerase bonds in proteins and is crucial for protein folding and response to endoplasmic reticulum (ER) stress (Zfin.org—ZFIN ID: ZDB-GENE-040426-705).

#### Karyopherin alpha 2 (KPNA2)

**Fig. 4:**
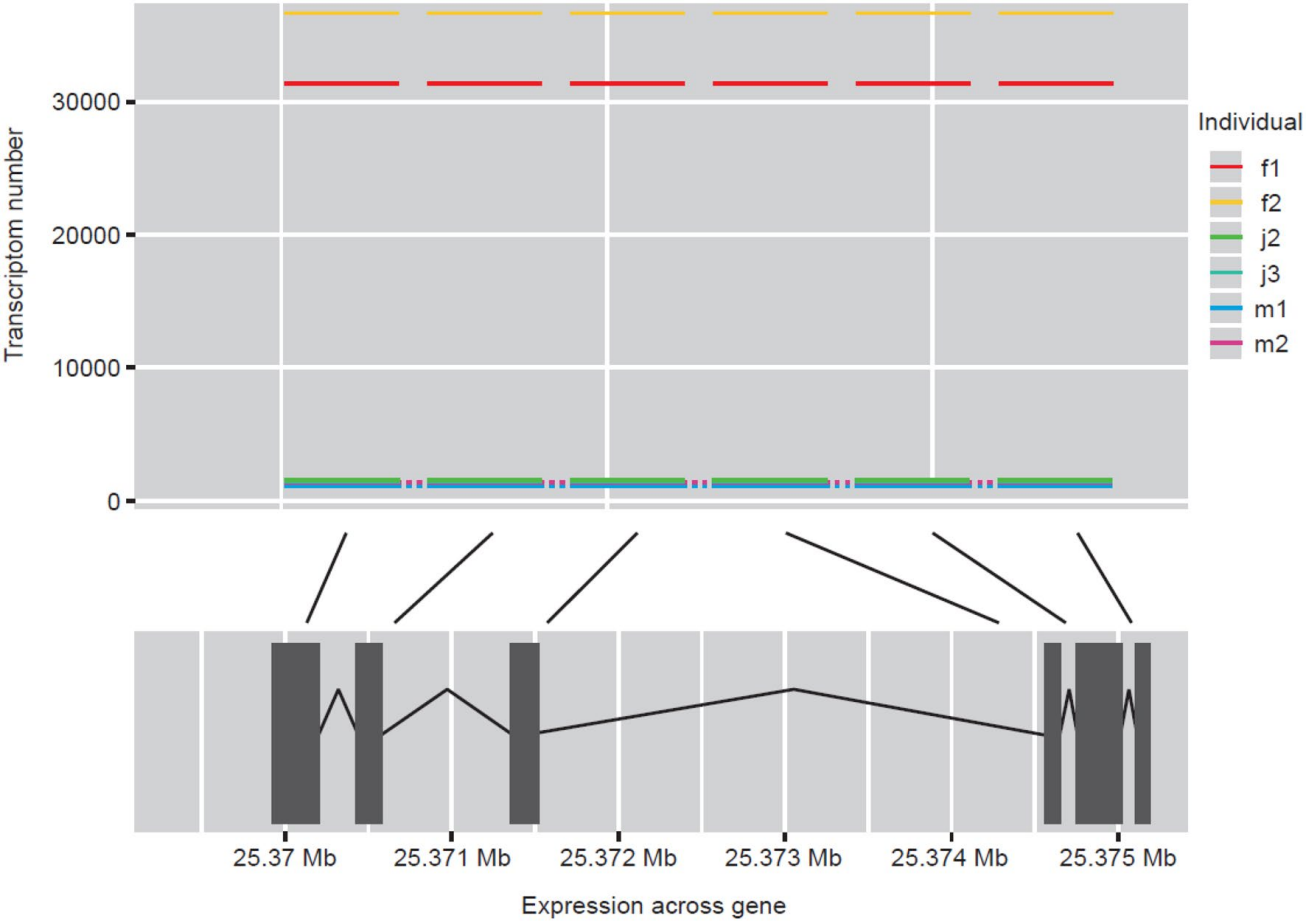
The relative expression of the female gene, kpna2, and the point of expression across the gene for males, females and juveniles for comparison

Our transcriptome data provides evidence for a higher expression of KPNA2 in adult female zebrafish compared with that of males.; Adult female transcriptome data is 27 times that of males with juvenile expression matching that of the adult male (Figs. 3, 4). KPNA2 is a protein-coding gene, involved in nuclear import signal receptor activity and protein import into the nucleus. Diseases associated with the gene include ovarian endodermal sinus tumour and ovarian primitive germ cell tumour (Zfin.org—ZFIN ID: ZDB-GENE-040718-22).

#### Cyclin B1 similar (ccnb1)

Our transcriptome data shows on average a 90-fold and 103-fold higher expression of ccnb1 in female zebrafish compared to males and juveniles, respectively (Fig. 3). Ccnb1 is predicted to contribute to protein kinase activity and is involved in oocyte maturation. Human orthologs of this gene are implicated in breast and prostate cancer (Zfin.org—ZFIN ID: ZDB-GENE-000406-10).

#### Cathepsin La (ctsla)

In our study, transcriptome data shows a 4.5-fold and 4-fold increase in expression in adult female zebrafish compared to males and juveniles, respectively (Fig. 3). Ctsla is speculated to have cysteine-type endopeptidase activity and thought to be involved in proteolysis (Zfin.org—ZFIN ID: ZDB-GENE-030131-106).

#### Bone morphogenetic protein (bmp15)

In this study, transcriptome expression of bmp15 is 352 and 2228 times higher in adult female zebrafish compared to males and juveniles, respectively (Fig. 3). Bmp15 is predicted to have cytokine and transforming growth factor receptor binding activity. Human orthologs of this gene are implicated in ovarian dysgenesis 2 and premature ovarian failure (Zfin.org—ZFIN ID: ZDB-GENE-030131-6115).

#### Zona pellucida glycoprotein 3 (zpb3)

Female transcriptome data has a 1.5 times higher expression of zpb3 in adult female zebrafish compared with males (Fig. 3). In addition, zpb3 is 1.8 times lower in females compared to juveniles, 28 dpf (Fig. 3). Zpb3 gene expression is partly co-ordinated by folliculogenesis-specific basic helix-loop-helix, transcription factor (Fig α), which is needed for female development (Zfin.org—ZFIN ID: ZDB-GENE-031121-1).

#### Chr4

We found chr4 is more highly expressed in females, with a 240-fold and 60-fold increase in transcriptome data seen in female compared with male and juvenile zebrafish, respectively (Fig. 3).

### Genes selected for early male sex-determination

One gene from the PCR in this study, two genes from high differentiation between male and females in NGS transcriptome data and one additional gene discovered from NGS data, all supported by previous literature, were selected as potential markers of male sex-determination in zebrafish, 28 dpf.

#### sox9a

In this study, PCR analysis showed that Sox9a had a higher expression in males when compared to females (see S4 and Figure 6, supporting information). Additionally, on average sox9a was expressed 2.5-fold and 3-fold higher in adult male and juvenile zebrafish compared to females based on NGS transcriptome data (Fig. 2); the strong expression of sox9a at juvenile stage is also seen in previous research. Sox9a exhibits chromatin-binding activity and is involved in cartilage and embryonic morphogenesis and glial cell differentiation (Zfin.org—ZFIN ID: ZDB-GENE-001103-1).

#### Glyceraldehyde-3-phosphate dehydrogenase, spermatogenic (gapdhs)

**Fig. 5:**
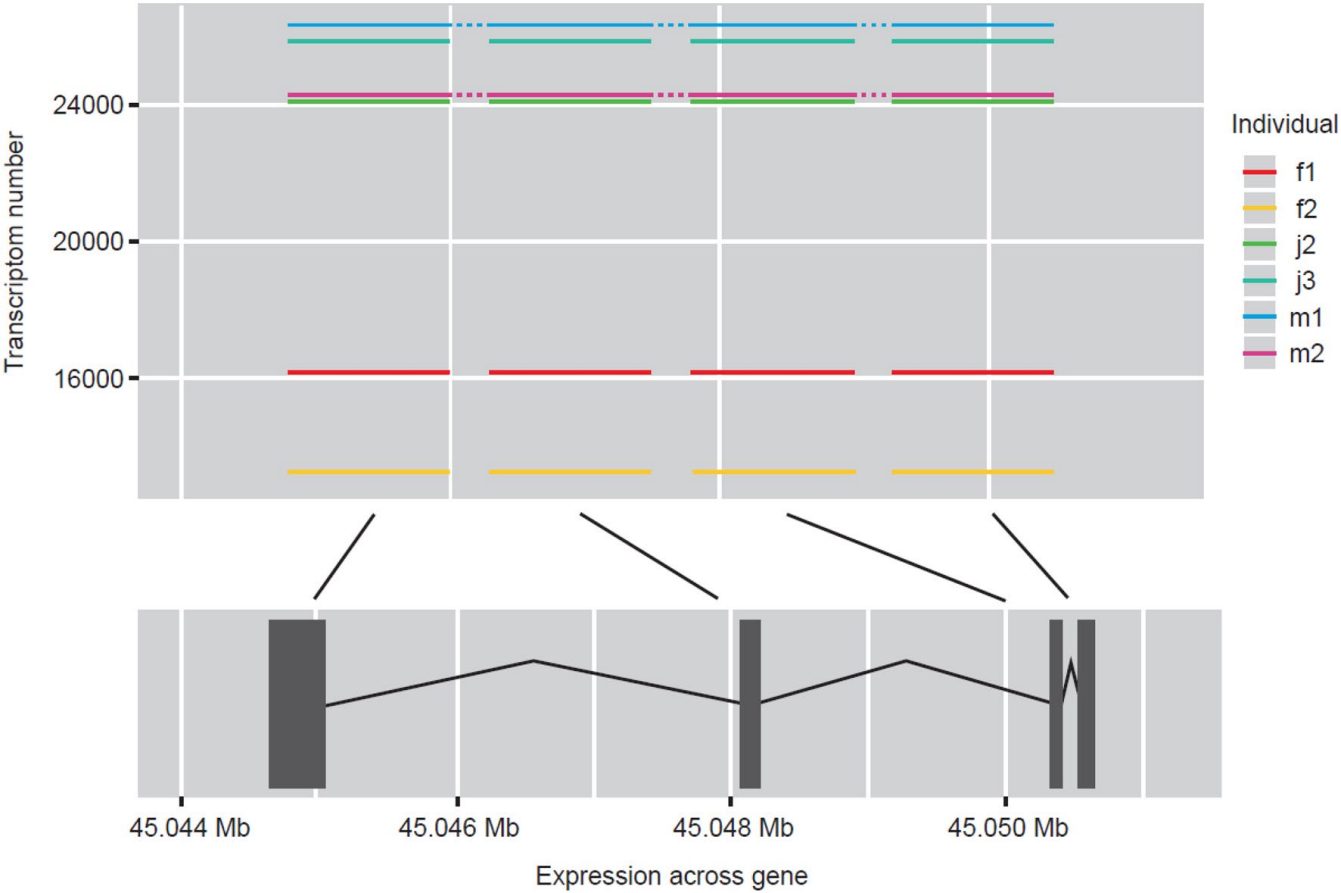
The relative expression of male gene, gapdhs, and the point of expression across the gene for males, females and juveniles for comparison

In this study, expression level of gapdhs was 1.7-fold higher in adult male zebrafish compared with females based on NGS transcriptome analysis (Fig. 2). We found gapdhs expression to be high in juveniles and adult male zebrafish; the average transcriptome number was almost identical (Fig. 5). Glyceraldehyde 3-phosphate dehydrogenase is an enzyme of the glycolytic pathway that is known to catalyse glyceraldehyde 3-phosphate to 1,3-bisphosphoglycerate. Gapdhs is expressed in several structures, including the cardiovascular system, digestive system, nervous system, neural tube,; and trigeminal placode (Zfin.org—ZFIN ID: ZDB-GENE-020913-1).

#### ATPase Na/K transporting, beta 1a polypeptide (atp1b1a)

A two-fold increase in both male and juvenile zebrafish expression can be seen in transcriptome data for atp1b1a compared to adult female zebrafish (Fig. 2). Atp1b1a is predicted to contribute to sodium–potassium-exchanging ATPase activity and is involved in several processes, including establishment or maintenance of epithelial cell apical polarity, regulation of cardiac muscle cell action potential and skin epidermis development (Zfin.org—ZFIN ID: ZDB-GENE-001127-3).

#### Cytochrome P450, family 26, subfamily b, polypeptide 1 (cyp26b1)

NGS data revealed sex-specific expression of cyp26b1, with 2.1 times more transcripts in adult male zebrafish compared with females as well as a 1.5-fold higher expression in juvenile zebrafish (Fig. 2). Cyp26b1 is predicted to have RA 4-hydroxylase activity and is involved in several processes, including animal organ morphogenesis, negative regulation of RA receptor signalling pathway and RA catabolic process. It is expressed in several zebrafish structures, including the brain; gill; hindbrain neural keel; pleuroperitoneal region; and skeletal system, as well as in testis (Zfin.org—ZFIN ID: ZDB-GENE-030131-2908).

## Discussion

### Expression analysis of sex-determining genes

The reference genome that we used for alignment of NGS data used the latest zebrafish sequence for the strain, TU, which should be sufficient as the reference genome to highlight relevant genes. However, previous literature states that there can be high variation in gene expression between strains (Guryev et al. 2006; Whiteley et al. 2011; Van Den Bos et al. 2017; Holden and Brown 2018). This should therefore be considered when primer and probes of selected sex-determination genes are developed and tested.

PCR results and NGS transcriptome data revealed several sex dominant genes that are also expressed in zebrafish, 28 dpf, results of which are also supported by previous research. The pattern of gene expression of the two adult male and two adult female zebrafish in comparison to the two juveniles indicates both juveniles are most likely males, as the transcriptome data of juveniles consistently reflects that of the adult males. Our gene selection highlighted many more female sex-specific genes compared to males; this matches previous literature which states that female-dominant genetic factors are necessary to determine zebrafish sex (Tong et al. 2010).

### Genes selected for early female sex-determination

#### gyg1a

Gyg1a is expressed in zebrafish at cleavage-16-cell-stage, 1.5–1.75 hpf, until hatching-long-pec-stage, 48–60 hpf, as well as at larval-stage, 14–20 dpf and juvenile stage, 30–44 dpf (Zfin.org—ZFIN ID: ZDB-GENE-040426-2910). Gyg1a has been confirmed as a female-enriched gene in previous studies (Van Maanen et al. 1999; Wen et al. 2005). Evidence from NGS data here and previous reports in literature taken as a whole is enough to support gyg1a as a gene for early female sex-determination in zebrafish.

#### rdh10b

Rdh10b is involved in RA biosynthetic and retinol processes. RA is present in female mice but absent in males (Koubova et al. 2006). Rdh10b is most highly expressed in the female gonad of zebrafish [bgee.org—gene: rdh10b—ENSDARG00000012369—*Danio rerio* (zebrafish)]. Retinoic mechanisms are central for tipping the sexual fate of gonads towards the female pathway in zebrafish and decreased RA leads to male phenotype (Rodríguez-Marí et al. 2013). Genes associated with retinal synthesis are upregulated in the ovary with higher gene expression and synthesis of vitellogenins in the liver but also in the extra-hepatic tissues (Levi et al. 2012). The expression of rdh10b in early developmental stages of zebrafish, from blastula, 128-cell stage, 2.25–2.5 hpf to larval-stage, 5 dpf until adulthood, 90–730 dpf (Zfin.org—ZFIN ID: ZDB-GENE-030909-7), in addition to NGS results, point to the usefulness of rdh10b as an early genetic marker for female sex-determination.

#### pdia

Stress often causes a male-biased sex ratio in fish; pdia4, the protein product of an ER chaperones, is down-regulated, five-fold, due to ER function decrease under stress conditions (Zheng et al. 2018). Pdia is expressed most highly in the female gonad [bgee.org—gene: pdia4—ENSDARG00000018491—*Danio* *rerio* (zebrafish)] and at 30–44 dpf in the ovaries of zebrafish (Miao et al. 2017). Using NGS data, pdia is predicted to be a female sex-specific gene with strong potential for early sex-determination in zebrafish.

#### KPNA2

KPNA2 is expressed in 31 organs, with the highest expression level in cleaving embryo and fourth highest in female gonad. KPNA2 is thought to be involved in female sexual fate and is expressed in the developing oocyte (Ly-Huynh et al. 2011; Zuccotti et al. 2013; Mihalas et al. 2015). In a study, KPNA2 protein was up-regulated two-fold in the female gonad during very early development and throughout oocyte growth. KPNA2 is expressed around the time of sex differentiation as well as at day 6 and adult stages in female but not male zebrafish (Major et al. 2011). All things considered, KPNA2 was selected as an early developmental gene for female sex-determination.

#### ccnb1

Ccnb1 is expressed in 48 organs, including nervous system; oocyte; pectoral fin; trunk mesenchyme; and unfertilized egg, with highest expression level in blastula and third highest expression in the mature ovarian follicle [bgee.org—gene: ccnb1—ENSDARG00000051923—*Danio rerio* (zebrafish)]. Ccb1 is found in oocytes and needed for oocytes maturation and female sexual development (Kondo et al. 1997, 2001; Knoll-Gellida et al. 2006; Wang et al. 2007; Nagahama and Yamashita 2008; Yasuda et al. 2013; Kotani et al. 2013; Takahashi et al. 2014; Horie and Kotani 2016; Dingare et al. 2018; Takei et al. 2018; Yi et al. 2019). Ccnb1 is expressed in early zebrafish development (Yasuda et al. 2010; Horie and Kotani 2016). Therefore, it was highlighted in our study as an early female sex-determining gene.

#### ctsla

Ctsla is expressed in 32 organs including the blood, gill, liver, pleuroperitoneal region and yolk syncytial layer, with highest expression level in gastrula at five stages of development and fourth highest expression in the mature ovarian follicle at fully-formed stage (Zfin.org—ZFIN ID: ZDB-GENE-030131-106). Ctsla shows different patterns of expression during embryogenesis and in adult zebrafish tissue. Levels of gene expression increase throughout development with highest ctsla accumulation in the adult ovary and oocytes. In zebrafish, ctsla is first detected in blastomers and later is localized in yolk syncytial layer cells. It is involved in yolk processing during oogenesis and embryogenesis. Ctsla mRNA is maternally inherited in zebrafish suggesting its vital role in early embryos (Tingaud-Sequeira and Cerdà 2007; Tingaud-Sequeira et al. 2011). For these reasons, ctsla was selected as a gene for early female sex-determination in zebrafish.

#### bmp15

Bmp15 is expressed in several structures, including the brain, digestive system, gonad, heart, and steroid hormone secreting cells, with the highest expression in the blastula and second highest expression in the female gonad at three stages, fully-formed, life-cycle and juvenile stage, 30–44 dpf [Zfin.org—ZFIN ID: ZDB-GENE-030131-6115; bgee.org—gene: bmp15—ENSDARG00000037491—*Danio rerio* (zebrafish)]. Bmp15 aids female sex differentiation, negative regulation of oocyte development and oocyte maturation. In zebrafish, bmp15 plays a crucial role in regulating functions of the gonad, it is found in oocytes and aids their maturation (Clelland et al. 2006; Yan et al. 2017). Bmp15 promotes female development; it increases expression of downstream gene cyp19a1a, an aromatase enzyme which converts androgens to oestrogens (Hosseini et al. 2019). The resultant increase in oestrogens creates differentiation of granulosa cells from bi-potential somatic cells. Loss or down-regulation of bmp15 in adult zebrafish results in disruption of ovarian development and a female-to-male sex reversal (Dranow et al. 2016; Crowder et al. 2018a; Hosseini et al. 2019), indicating it as a marker for early female sex-determination in zebrafish.

#### zpb3

Fig alpha, fig α, influences zpb3 gene expression and is needed for female development (Zfin.org—ZFIN ID: ZDB-GENE-031121-1). In mammals, Fig α is a germ cell-specific transcription factor, which is necessary for normal formation of the ovarian follicle and oocytes (Schlessinger et al. 2011). Fig α is expressed throughout zebrafish development with the peak of both fig α and zpb expression coinciding with the start of zebrafish gonad differentiation at 22 dpf. There is another peak of expression at 25 dpf when the onset of gonadal differentiation to ovary in female zebrafish is expected. The highest expression of zpb is in the female gonad and seventh highest in mature ovarian follicle (Jørgensen et al. 2008) [bgee.org—gene: zp3c—ENSDARG00000092919—*Danio rerio* (zebrafish)]. Fig α is thought to be crucial in oocyte preservation, normal ovarian development in mice and is an oocyte specific marker in medaka (Soyal et al. 2000; Kanamori et al. 2006). In zebrafish, low expression and down-regulation of fig α is seen just before oocyte apoptosis at 19–20 dpf in individuals developing as male. Fig α and related female-fated genes are highly expressed after 22 dpf to adulthood (Jørgensen et al. 2008). This information taken with our data and the fact that zpb3 is expressed in early development provides evidence for the use of zpb3 as an early marker of adult zebrafish sexual fate.

#### Chr4

In previous research, a strongly sex-linked locus was found only at the end of the long right arm of Chr4 in natural zebrafish populations. Chr4R lacks protein-coding genes, contains most of the genome’s 5S–RNA genes, is enriched in satellite repeats and has high GC content (Anderson et al. 2012; Howe et al. 2013); in other species, such as mammals, these traits are linked to sex chromosomes (Charlesworth et al. 2005). Sex-linked markers found only on the chromosome arm with cytogenetic properties of sex chromosomes indicates that Chr4R is a sex chromosome in zebrafish. Another study found that two domesticated strains, AB and TU, lacked this sex-linked loci and thus it was concluded that this female sex-linked region had been lost in domesticated strains. It was thought that domestication led to new methods of sex-determination through selection or uncovering of minor genetic sex-determining mechanisms (Wilson et al. 2014). However, in our study we concluded that Chr4 was more highly expressed in adult female zebrafish compared with males, despite being from the domesticated strain, AB.

### Genes selected for early male sex-determination

#### Sox9a

Sox9a is expressed in 76 organs, including brain, ectoderm, head, reproductive system and skeletal system, with highest expression level in ceratohyal cartilage at five stages of early development. This pattern of male-biased sex-expression in Sox9a can be seen in literature where it is reported to be predominantly expressed in the testis (Groh et al. 2011). Many other studies support these findings that sox9a is a male sex-determining gene (Chiang et al. 2001; Vidal et al. 2001; Gasca et al. 2002; Klüver et al. 2005; Rodríguez-Marí et al. 2005; Jørgensen et al. 2008; Tong et al. 2010; Major et al. 2011; Sun et al. 2013; Liu et al. 2013; Chen et al. 2017; Lin et al. 2017b; Yu et al. 2018; Crowder et al. 2018a). Sox9a is expressed in early zebrafish development from 0–44 dpf (Jørgensen et al. 2008), in the testis at juvenile stage, 30–44 dpf, and in fully developed adult stages (Zfin.org—ZFIN ID: ZDB-GENE-001103-1). Our findings from PCR and NGS together with previous literature highlight sox9a as a gene for male sex-determination in early zebrafish development.

#### gapdhs

Glyceraldehyde-3-phosphate dehydrogenase spermatogenic, gapdhs, is a testis-specific enzyme encoded by gapdhs gene and is required for sperm motility and male fertility (Welch et al. 1995; Kuravsky et al. 2011; Fujihara et al. 2019). Gapdhs encodes a protein, which has an important role in the carbohydrate metabolism. Gapdhs is necessary for energy production during spermatogenesis and in the spermatozoon and is tenth highest expressed in the testis of zebrafish [bgee.org—gene: gapdhs—ENSDARG00000039914—*Danio* *rerio* (zebrafish)]. In mice, gapdhs is first expressed at 20 dpf in the post-meiotic germ cells. Expression levels increase until 24 dpf and then remain constant during maturity. After sexual maturation at 120 dpf, gapdhs protein is in both sertoli cells and elongated sperms (Liu et al. 2013). Gapdhs-knock-out mice had impaired sperm motility, higher levels of infertility and a lower ATP concentration; 10.4% that of wild-type mice. Anti-gapdhs antibodies were also able to inhibit zona pellucida penetration (Miki et al. 2004; Liu et al. 2013; Takei et al. 2014; Paoli et al. 2016). Gapdhs is one of the two isoforms of this enzyme in mammals and is found only in sperm (Paoli et al. 2016). From our results, we can see that gapdhs is expressed in early zebrafish development therefore it is ideal for use as a male sex-determination gene marker for zebrafish, 28 dpf.

#### atp1b1a

Atp1b1a is expressed in 39 organs, including the cardiovascular system, epithelium, mesoderm, renal system and sensory system, with highest expression level in the kidney and seventh highest expression in the testis at fully-formed stage [Zfin.org—ZFIN ID: ZDB-GENE-001127-3; bgee.org—gene: atp1b1a—ENSDARG00000013144—*Danio rerio* (zebrafish)]. It is expressed in early development in zebrafish from gastrula 50%-epiboly-stage to adult-stage (Ma and Jiang 2007; Wang et al. 2008; Abbas and Whitfield 2009; Hatzold et al. 2016) (Zfin.org—ZFIN ID: ZDB-GENE-001127-3). 17-Alpha-ethinyloestradiol (EE2) can be found in sewage effluent at concentrations that change normal reproductive function in fish. Atp1b1a was down-regulated with EE2 exposure (Martyniuk et al. 2007). All things considered, atp1b1a was selected as marker gene for early male sex-determination.

#### cyp26b1

The balance of RA is related to sex-determination and is necessary for spermatogenesis. The RA degrading enzyme cyp26b1 has differential expression in the gonads with higher expression in the testis (Kashimada et al. 2011; Kipp et al. 2011; Bowles et al. 2016). Testis and ovary samples in zebrafish, 90 dpf, are sexually dimorphic with higher expression of cyp26b1 in the testis (Pradhan and Olsson 2015). During mouse gonadogenesis, up-regulation of cyp26b1 expression in male-fated individuals leads to loss of RA and protects germ cells from entering into meiosis in developing testes, while female-specific down-regulation of cyp26b1 expression allows RA to induce germ cells to enter into meiosis in embryonic ovaries (Rodríguez-Marí et al. 2013).

Cyp26b1 and transcription factor genes, sf1 and sox9, are co-expressed in sertoli and leydig cells in mouse foetal testes. Sf1 and sox9 expression ensure germ cells become male by activating and up-regulating cyp26b1 in mice. In addition to this, foxl2 acts as an antagonist of cyp26b1 and is expressed in ovary-fated gonads; knock-out of the ovarian transcriptome factor, foxl2, increases cyp26b1 expression 20-fold in XX mice gonads in relation to wild-type individuals (Kashimada et al. 2011). This indicates cyp26b1 as a useful gene for early sexual fate in zebrafish.

## Conclusions

The identification of the early expression of these male- and female-specific genes will have profound implications in aiding future developmental, biomedical, toxicological, eco-toxicological and genetic zebrafish research. This in turn is useful for human pharmaceutical drug discovery and trials, and toxicity testing, as sex-related, covered, co-founding factors are removed. More research should now be carried out to allow us to clarify the ability of these selected genes to determine sex at 28 dpf with the development of TaqMan qPCR assays for the novel selected genes. With these early markers, improved sex determination in juvenile zebrafish will soon be achievable; in turn allowing improvements in human health and environmental trials.

## Electronic supplementary material

Below is the link to the electronic supplementary material.

Supplementary file1 (DOCX 96 kb)
