# Supplementary material for: Shedding new light on early sex determination in zebrafish
Source: Arch Toxicol. 2020 Sep 25;94(12):4143–58. doi: 10.1007/s00204-020-02915-y (PMC7655572; doi:10.1007/s00204-020-02915-y)
Supplement: Supplementary file 1 — Supplementary file1 (DOCX 96 kb) [file 204_2020_2915_MOESM1_ESM.docx]

**Supplementary material**

**S1**

**Table 1: Details of zebrafish developmental stages**

| **Period** | **Stage** | **Begins** | **Developmental Landmarks** |
| --- | --- | --- | --- |
| **Zygote (0 - 0.75 h)** | 1-cell | 0.00 h | Cytoplasm streams toward animal pole to form blastodisc |
| **Cleavage** | 2-cell | 0.75 h | Partial cleavage |
| **(0.75 - 2.25 h)** | 4-cell | 1.00 h | 2 X 2 array of blastomeres |
|  | 8-cell | 1.25 h | 2 X 4 array of blastomeres |
|  | 16-cell | 1.50 h | 4 X 4 array of blastomeres |
|  | 32-cell | 1.75 h | 4 X 8 array of blastomeres |
|  | 64-cell | 2.00 h | 3 regular tiers of blastomeres |
| **Blastula** | 128-cell | 2.25 h | 5 blastomere tiers; cleavage planes irregular |
| **(2.25 - 5.25 h)** | 256-cell | 2.50 h | 7 blastomere tiers |
|  | 512-cell | 2.75 h | 9 blastomere tiers; YSL forms |
|  | 1k-cell | 3.00 h | 11 blastomere tiers; single row of YSL nuclei; asynchronous cell cycle |
|  | High | 3.33 h | > 11 blastomere tiers; blastodisc flattening begins; YSL nuclei in two rows |
|  | Oblong | 3.66 h | Blastodisc flattening; multiple rows of YSL nuclei |
|  | Sphere | 4.00 h | Spherical shape; flat border between blastodisc and yolk |
|  | Dome | 4.33 h | Yolk cell bulging toward animal pole as epiboly begins |
|  | 30%-epiboly | 4.66 h | Blastoderm an inverted cup of uniform thickness |
| **Gastrula** | 50%-epiboly | 5.25 h | Blastoderm remains of uniform thickness |
| **(5.25 - 10.33 h)** | Germ-ring | 5.66 h | Germ ring visible from animal pole; 50%-epiboly |
|  | Shield | 6.00 h | Embryonic shield visible from animal pole; 50%-epiboly |
|  | 75%-epiboly | 8.00 h | Dorsal side distinctly thicker; epiblast, hypoblast, evacuation zone visible |
|  | 90%-epiboly | 9.00 h | Axis and neural plate; brain and notochord rudiments |
|  | Bud | 10.00 h | Tail bud prominent; early polster; 100%-epiboly |
| **Segmentation** | 1-4 somites | 10.33 h | First somite furrow |
| **(10.33 - 24 h)** | 5-9 somites | 11.66 h | Polster prominent; optic vesicle, Kupffer's vesicle, neural keel |
|  | 10-13 somites | 14 h | Pronephros forms |
|  | 14-19 somites | 16 h | EL (embryo length) = 0.9 mm; otic placode, brain neuromeres |
|  | 20-25 somites | 19 h | EL = 1.4 mm; lens, otic vesicle, hindbrain neuromeres |
|  | 26+ somites | 22 h | EL = 1.6 mm; blood islands, otoliths, midbrain-hindbrain boundary |
| **Pharyngula** | Prim-5 | 24 h | EL = 1.9 mm; early pigmentation, heartbeat |
| **(24 - 48 h)** | Prim-15 | 30 h | EL = 2.5 mm; early touch reflex, retina pigmented |
|  | Prim-25 | 36 h | EL = 2.7 mm; early motility, tail pigmentation |
|  | High-pec | 42 h | EL = 2.9 mm; rudiments of pectoral fins |
| **Hatching** | Long-pec | 48 h | EL = 3.1 mm; elongated pectoral fin buds |
| **(48 - 72 h)** | Pec-fin | 60 h | EL = 3.3 mm; pectoral fin blades |
| **Larval** | Protruding-mouth | 72 h | 3.5 mm total body length |
|  | Day 4 | 96 h | 3.7 mm total body length |
|  | Day 5 | 120 h | 3.9 mm total body length; 6 teeth |
|  | Day 6 | 144 h | 4.2 mm total body length |
|  | Days 7-13 | 168 h | 4.5 mm total body length; 8 teeth |
|  | Days 14-20 | 14 d | 6.2 mm total body length; 10 teeth |
|  | Days 21-29 | 21 d | 7.8 mm total body length |
| **Juvenile** | Days 30-44 | 30 d | 10 mm total body length; adult fins/pigment |
|  | Days 45-89 | 45 d | 14 mm total body length; 12 teeth |
| **Adult (90 d - 2 y)** |  | 90 d | Breeding adult |

**S2**

**Table 2: Overview of genes used for the development of the gene expression assay**

| Protein / Gene | Abbreviation | NCBI RefSeq | Reason for selection | Reference |
| --- | --- | --- | --- | --- |
| Actin, beta 1 | *actb1* | NM_131031.1 | Reference gene |  |
| SRY (sex determining region Y)-box 9a | *sox9a* | NM_131643.1 | Possible contribution to sex determination | Yan et al. 2002; Dutton et al. 2009 |
| SRY (sex determining region Y)-box 9b | *sox9b* | NM_131644.1 | Possible contribution to sex determination | Huang et al. 2016 |
| Vitellogenin 1 | *vtg1* | NM_001044897.3 | Change in gene expression of vtg1 after exposure to androgens | Belt et al. 2003; Örn et al. 2003; Fent et al. 2018 |
| Cytochrome P450, family 19, subfamily A, polypeptide 1a | *cyp19a1a* | NM_131154.3 | Female gonadal and sexual development | Lau et al. 2016; Chen et al. 2017 |
| Cytochrome P450, family 19, subfamily A, polypeptide 1b | *cyp19a1b* | NM_131642.2 |  |  |
| Double-sex and mab-3 related transcription factor 1 | *dmrt1* | NM_205628.1  NM_205761.1 | Gonadal and sexual development | Lin et al. 2017; Webster et al. 2017a |
| Nuclear receptor subfamily 0, group B, member 1 | *nr0b1 (dax1)* | NM_001082947.1 | Negative regulation of male gonadal development | Chen et al. 2016 |
| Cytochrome P450, family 17, subfamily A, polypeptide 1 | *cyp17a* | NM_212806.3 | Progesterone metabolism | Pallan et al. 2015 |
| Insulin-like growth factor 3 | *igf3* | NM_001115050.1 | Sperm capacitation and germ cell development | Li et al. 2011; Lemos et al. 2017; Lin et al. 2017 |

**S3**

**Table 3: Overview of the seven developed primers used for sex-specific expression analysis**; including primer and probe sequences, melting temperatures and amplicon length

| **Gene** | **Name** | **5'-3' Sequenz** | **Temp [°C]** | **Amplicon length** |
| --- | --- | --- | --- | --- |
| *actb1* | actb1_FOR01 | ACAGAGAGAAGATGACACAGATCATG | 58 | 120bp |
|  | actb1_REV01 | AGTCCATCACAATACCAGTAGTACG | 58 |  |
|  | actb1_TaqMan01 | CAACACCCCTGCCATGTATGTGGCCATCCAG | 69 |  |
| *cyp17a1* | cyp17a1_FOR01 | TGAGGAACACAAGGTGACCTACAG | 59 | 109bp |
|  | cyp17a1_REV01 | GACATCACGAGTGCTGCTG | 58 |  |
|  | cyp17a1_TaqMan01 | CAGCGGGATCTCCTGGATGCTCTTCTGAGGG | 69 |  |
| *nr0b1* | nr0b1_FOR01 | GCCATTCTGTTCAACCCAGATGTTG | 60 | 146bp |
|  | nr0b1_REV01 | GCGAGGAAGAGTTTGGCGAATCTC | 61 |  |
|  | nr0b1_TaqMan01 | CCTGCAGAGCGAGGCGAACCAGGCAC | 70 |  |
| *sox9b* | sox9b-201_FOR01 | CTGGAGAACACTCCGGTCAGT | 60 | 153bp |
|  | sox9b-201_REV01 | CCACCGCACCGAAGTCAATG | 61 |  |
|  | sox9b_TaqMan01 | CCGCTTCAGATCCGCTTTACTGCACACCGGC | 70 |  |
| *vtg1* | vtg1_FOR03 | GTGATGCACCTGCCCAGATTG | 61 | 159bp |
|  | vtg1_REV03 | CCTTGAACTGAGACCAGATAGCCTC | 60 |  |
|  | vtg1_TaqMan03 | CAAGGAGGCAACACGCAAGAGCTGGACAAGC | 70 |  |
| *cyp19a1b* | cyp19a1b_FOR02 | CAGAGCTGATATTTGCTCAGAACCATG | 59 | 174bp |
|  | cyp19a1b_REV02 | ACTGTATCTCCTGTACGATTTGCTCTTC | 59 |  |
|  | cyp19a1b_TaqMan02 | CTGTGGACGACGTGAGGCAGTGTGTGCTG | 70 |  |
| *cyp19a1a* | cyp19a1a_FOR02 | GCGTGTGGAGCTACAGCTAC | 60 | 103bp |
|  | cyp19a1a_REV02 | AGAAAGAAGGACCTGGAATGTGTG | 59 |  |
|  | cyp19a1a_TaqMan02 | CTGCTGCTGCTACTCTGCCTGCTGCTGGC | 71 |  |

**S4**

**Gene expression analysis of adult zebrafish**

Before sample processing, the sex of the adult zebrafish was visually assigned based on typical characteristics. Significant differences could be seen in gene expression between individuals at a molecular level. Gene expression analysis showed gender-specific expression of cyp17a1, cyp19a1a, cyp19a1b, vtg1, igf3 and dmrt1 in Zebrafish, with the first 5 being female-fated genes and the final one a male-related gene (Fig. 6). For the genes cyp19a1b, nr0b1, sox9a and sox9b no gender-associated gene expression could be confirmed with the method used (Fig. 6).

**Fig. 6: Comparison of the delta Ct value of adult female and male zebrafish shown as a bar chart.** Blue: mean value of the ∆Ct value of the male gene expression, red: mean value of the ∆Ct value of the female gene expression. Significant differences can be seen in the genes cyp17a1, cyp19a1a, vtg1, igf3 and dmrt1. A low ∆Ct value corresponds to a strong expression, a high ∆Ct value to a weaker expression of the analysed gene. Significant differences in gene expression between sex were marked on the graph using different numbers of stars, where one star (*) for p ≤ 0.0332, two stars (**) for p ≤ 0.0021, three stars (***) for p ≤ 0.0002 and four stars (****) for p < 0.0001. The bars without stars in the graphs showed no significant differences (ns)

**S5**

**Table 4: Expression of selected genes from NGS data with the highest transcriptome differences between males and female;** indicated is the chromosome position, chromosome number, gene name, juvenile (J), female (F) and male (M) transcriptome expression and the difference in F and M transcriptome data

| **Position** | **Chromosome no.** | **Gene name/**  **description** | **juv2** | **juv3** | **f2** | **f1** | **m2** | **m1** | **Difff-m** |
| --- | --- | --- | --- | --- | --- | --- | --- | --- | --- |
| NC_007125.7:5,859,457-5,867,146 | 14 | tubulin, beta2c, beta 4B class Ivb | 42742.5 | 46015.7 | 232784.7 | 184827.3 | 37359.2 | 37037.0 | 171607.9 |
| NC_007127.7:17,190,671-17,199,601 | 16 | gapdh - Glyceraldehyde 3-phosphate dehydrogenase | 128155.7 | 114001.5 | 276314.7 | 206595.8 | 94834.8 | 144259.5 | 121908.1 |
| NC_007116.7:54,709,393-54,716,749 | 5 | ccnb1 - cyclin B1 similar | 658.2 | 627.4 | 81366.3 | 53898.2 | 648.9 | 860.1 | 66877.8 |
| NW_018394460.1:68,606-76,161 | 1 | actb1 - actin beta1 | 37509.7 | 19922.0 | 110611.3 | 142346.8 | 39905.9 | 83412.0 | 64820.1 |
| NC_007125.7:41,464,369-41,470,993 | 14 | MID1ip1 - interacting protein 1, like | 4172.6 | 4495.8 | 51340.5 | 43828.4 | 1954.6 | 4840.2 | 44187.1 |
| NC_007132.7:3,164,519-3,175,735 | 21 | hnrnpabb - heterogeneus nuclear ribonucleoprotein A/Bb | 7610.7 | 7621.0 | 53684.2 | 47559.6 | 11089.0 | 9775.6 | 40189.6 |
| NC_007115.7:16,827,111-16,837,904 | 4 | Ldhba - lactate dehydrogenase Ba | 16646.9 | 17678.7 | 57936.2 | 51922.4 | 12720.0 | 19697.9 | 38720.3 |
| NC_007129.7:171,603-196,844 | 18 | larp6 - La ribonuecleoprotein domain family, member 6 | 1558.8 | 1149.4 | 39958.7 | 36768.8 | 1883.5 | 2064.3 | 36389.9 |
| NC_007135.7:17,332,682-17,343,458 | 24 | pdia4 - protein disulfide isomerase family A, member 4 | 2560.9 | 2102.6 | 53010.5 | 40197.0 | 10579.2 | 13051.3 | 34788.5 |
| NC_007128.7:32,620,903-32,632,283 | 17 | ctsba, cathepsin Ba | 6282.6 | 6122.7 | 59859.9 | 27330.5 | 8766.1 | 11530.4 | 33447.0 |
| NC_007114.7:25,367,930-25,379,017 | 3 | kpna2 - karyopherin alpha 2(RAG cohort 1 importin alpha 1) | 1613.8 | 1423.3 | 36808.9 | 31501.7 | 1411.2 | 1057.5 | 32920.9 |
| NC_007120.7:424,345-435,915 | 9 | aln371 - basic leucine zipper and W2 domains 1b, transcript variant X1 | 3718.6 | 3385.3 | 53253.0 | 49075.8 | 18511.0 | 19603.6 | 32107.1 |
| NC_007132.7:30,770,188-30,789,414 | 21 | rrm1 - Ribonucleotide reductase M1 polypeptide | 1959.8 | 1912.9 | 37981.5 | 32345.7 | 2804.8 | 3729.3 | 31896.5 |
| NC_007116.7:44,804,375-44,812,915 | 5 | ctsla - cathepsin La | 10221.3 | 9344.2 | 42760.5 | 37063.7 | 11558.9 | 6157.4 | 31053.9 |
| NC_007121.7:29,768,231-29,793,466 | 10 | hyou1 - hypoxia up-regulated 1 | 2723.9 | 2644.9 | 39788.2 | 35874.9 | 8412.1 | 7351.0 | 29950.0 |
| NC_007128.7:1,358,044-1,371,344 | 17 | SIVA1, apoptosis-inducing factor | 216.9 | 232.3 | 31950.9 | 27199.3 | 354.0 | 432.3 | 29182.0 |
| NC_007113.7:24,939,232-24,946,976 | 2 | gyg1a - glycogenin 1a | 4155.6 | 3828.6 | 32685.2 | 29394.6 | 4574.8 | 1878.4 | 27813.3 |
| NC_007118.7:66,632,233-66,656,033 | 7 | aln315 | 5400.1 | 5068.6 | 29876.4 | 30896.8 | 6082.7 | 5453.0 | 24618.8 |
| NC_007117.7:42,375,911-42,390,579 | 6 | Sec61a1 - Sec61 translocon alpha 1 subunit, like | 1624.4 | 1656.5 | 28905.4 | 26419.2 | 3379.4 | 2945.7 | 24499.7 |
| NC_007121.7:40,819,804-40,828,678 | 10 | pcna - proliferating cell nuclear antigen | 7036.1 | 6845.5 | 34334.1 | 30935.2 | 7844.7 | 8529.5 | 24447.5 |
| NC_007113.7:30,180,669-30,188,307 | 2 | rdh10b - retinol dehydrogenase 10b | 465.6 | 390.5 | 24519.5 | 22451.1 | 589.7 | 630.6 | 22875.2 |
| NC_007112.7:22,645,756-22,654,024 | 1 | uchl1 - ubiquitin carboxyl-terminal esterase L1 (ubiquitin thiolesterase) | 1007.4 | 1133.7 | 23909.3 | 22123.7 | 1276.2 | 1519.1 | 21618.9 |
| NC_007125.7:33,472,669-33,483,527 | 14 | lamp2 - lysosomal-associated membrane protein 2, transcript variant X1 | 1244.5 | 1196.6 | 25539.9 | 22311.8 | 2223.9 | 2537.5 | 21545.2 |
| NC_007113.7:3,500,357-3,511,255 | 2 | zgc:92744 - stress associated endoplasmatic reticulum protein 1 | 5737.7 | 4894.7 | 30285.1 | 26097.6 | 6976.9 | 6490.1 | 21457.9 |
| NC_007130.7:47,973,912-47,999,424 | 19 | zgc158376 | 9902.8 | 6934.3 | 30671.3 | 30237.4 | 9994.3 | 10236.3 | 20339.1 |
| NC_007122.7:5,466,769-5,492,184 | 11 | cse1 - chromosome segregation 1-like (yeast) | 1901.6 | 2128.5 | 23509.0 | 21498.0 | 3412.1 | 3356.7 | 19119.1 |
| NC_007117.7:57,476,337-57,487,411 | 6 | Snrpb - Small nuclear ribonucleoprotein polypeptides B and B1 | 5880.5 | 5330.5 | 25080.4 | 26584.0 | 7199.0 | 6405.6 | 19029.9 |
| NC_007133.7:14,115,091-14,128,065 | 22 | bzw1a - basic leucine zipper and W2 domains 1a | 4975.7 | 4678.1 | 25114.3 | 22508.0 | 5306.0 | 4883.8 | 18716.3 |
| NC_007122.7:3,891,267-3,909,982 | 11 | rpn1 - ribophorin1 | 7358.9 | 5893.2 | 27660.9 | 28944.2 | 8958.6 | 10520.9 | 18562.8 |
| NC_007133.7:5,631,811-5,656,783 | 22 | mcm2 - minichromosome maintenance complex component 2 | 3380.0 | 3123.4 | 20212.6 | 20315.0 | 2806.4 | 4273.6 | 16723.8 |
| NC_007134.7:44,234,249-44,253,006 | 23 | 7SK snRNA methylphosphate capping enzyme-like | 1421.2 | 1128.1 | 16406.0 | 17766.8 | 1039.6 | 1082.4 | 16025.4 |
| NC_007120.7:46,403,587-46,417,813 | 9 | connexin 43.4 | 1256.1 | 1225.3 | 18214.2 | 16840.4 | 1750.0 | 1700.6 | 15802.0 |
| NC_007115.7:25,171,218-25,183,582 | 4 | atp5c1 - ATP synthase, H transporting, mitochondrial F1 complex, gamma poly peptide 1, transcript variant 3 | 24065.0 | 22701.1 | 37739.0 | 31094.2 | 17096.6 | 21282.8 | 15226.9 |
| NC_007136.7:5,981,448-5,992,182 | 25 | ppib - Peptidylprolyl Isomerase B (cyclophilin B) | 22273.4 | 21291.6 | 28553.0 | 29588.5 | 15029.3 | 13592.0 | 14760.1 |
| NC_007114.7:55,096,457-55,106,310 | 3 | hbaa1 - hemoglobin, alpha adult 1 | 24585.6 | 21781.2 | 11987.8 | 15467.0 | 24546.6 | 21273.0 | -9182.4 |
| NC_007127.7:45,042,642-45,071,814 | 16 | gapdhs - glyceraldehyde-3-phosphate dehydrogenase, spermatogenic | 24122.1 | 25901.3 | 13281.5 | 16172.8 | 24304.4 | 26333.8 | -10591.9 |
| NC_007117.7:29,215,445-29,229,487 | 6 | atp1b1a - ATPase Na/K transporting, beta 1a polypeptide | 23058.6 | 23810.7 | 9477.7 | 13055.8 | 23207.3 | 24501.6 | -12587.7 |
| NC_007119.7:54,074,192-54,079,740 | 8 | rho - rhodopsin | 52907.8 | 47605.7 | 16620.2 | 29225.1 | 40721.8 | 31552.9 | -13214.7 |
| NC_007129.7:26,094,785-26,151,705 | 18 | ankrd34c - protein coding | 40391.2 | 40627.8 | 24508.3 | 28493.7 | 44490.3 | 41370.2 | -16429.3 |
| NC_007117.7:41,094,028-41,150,940 | 6 | slc613a - solute carrier family 6 - FK506 binding protein 5, transcript variant X2 | 765.1 | 1519.6 | 12558.5 | 13753.5 | 30896.9 | 43027.2 | -23806.1 |
| NC_007128.7:115,110-126,665 | 17 | actc1b - actin, alpha and cardiac muscle 1b | 2227693.1 | 1924506.4 | 142666.5 | 190058.5 | 252348.1 | 144482.8 | -32053.0 |
| NC_007133.7:37,886,141-37,900,579 | 22 | fetub - fetuin b | 197484.9 | 133008.2 | 41441.4 | 48677.6 | 124708.1 | 37761.0 | -36175.1 |
| NC_007118.7:7,046,245-7,135,368 | 7 | actn3b - actinin alpha 3b, transcript variant X1 | 72592.8 | 65082.6 | 20921.5 | 26481.9 | 97856.2 | 42677.7 | -46565.2 |
| NC_007112.7:7,544,347-7,556,358 | 1 | mylz3 - myosin, polypetide 3 and skeletal muscal | 582814.5 | 577933.8 | 55912.4 | 68229.9 | 151183.7 | 78913.3 | -52977.4 |
| NC_007131.7:31,267,702-31,286,894 | 20 | apobb1 - Apolipoprotein Bb, tandem duplicate 1, transcript variant X1 | 140691.4 | 161111.1 | 53647.6 | 55242.6 | 80683.7 | 154533.2 | -63163.3 |
| NC_007127.7:23,976,978-23,982,268 | 16 | apoa2 - Apolipoprotein A2 | 262740.1 | 225511.7 | 119227.9 | 112684.0 | 241948.7 | 177242.6 | -93639.7 |
| NC_007126.7:23,641,718-23,649,073 | 15 | ckmb - creatine kinase muscle b | 555951.6 | 510368.3 | 215503.5 | 230292.6 | 667206.2 | 374258.7 | -297834.4 |

**S6**

**Table 5: F genes selected for their role in early sex-determination,** including highest difference of M and F transcriptome data, PCR from this study and additional genes from NGS data. Indicated is the chromosome position, chromosome number, gene name, J, F and M transcriptome expression, the difference in F and M transcriptome data, previous evidence of J and sex-specific expression

| **Chromosome Position** | **Chromosome no.** | **Gene name and description** | **juv2** | **juv3** | **f2** | **f1** | **m2** | **m1** | **Diff f-m** | **Juvenilles expression** | **Papers Sex determination genetic differences** |
| --- | --- | --- | --- | --- | --- | --- | --- | --- | --- | --- | --- |
| NC_007113.7:24,939,232-24,946,976 | 2 | gyg1a - glycogenin 1a | 4155.6 | 3828.6 | 32685.2 | 29394.6 | 4574.8 | 1878.4 | 4155.6 | Cleavage:16-cell (1.5h-1.75h) to Hatching:Long-pec (48.0h-60.0h) (zfin.org - ZFIN ID: ZDB-GENE-040426-2910) Larval:Days 14-20, Juvenile:Days 30-44 (Uniprot.org - UniProtKB - Q803Q1 (Q803Q1_DANRE)) | C. Wen et al., 2005, M.V Maanen, P A Fournier, T N Palmer, L.J Abraham,, 1999 |
| NC_007113.7:30,180,669-30,188,307 | 2 | rdh10b - Retinol dehydrogenase 10b | 465.6 | 390.5 | 24519.5 | 22451.1 | 589.7 | 630.6 | 465.6 | Blastula:128-cell (2.25h-2.5h) to Adult (90d-730d, breeding adult) (zfin.org - ZFIN ID: ZDB-GENE-030909-7) Larval:Day 5 (Uniprot.org - UniProtKB - Q7T2D1 (RD10B_DANRE)) | Rodríguez-Marí et al., 2013; L. Levi, T. Ziv, A. Admon, B. Levavi-Sivan, E. Lubzens, 2012 |
| NC_007135.7:17,332,682-17,343,458 | 24 | pdia - Protein disulfide isomerase family A, member 4 | 2560.9 | 2102.6 | 53010.5 | 40197.0 | 10579.2 | 13051.3 | 2560.9 | Larval:Day 5 ([bgee.org/](https://bgee.org/) - Gene: pdia4 - ENSDARG00000018491 - Danio rerio (zebrafish)), Translation repression by maternal RNA binding protein zar1 is essential for early oogenesis in zebrafish - At days 30-44 pdia4 expressed in ovaries (Miao et al., 2017) | B. Thisse, 2004, Miao et al., 2017, M. Zheng, J. Lu and D. Zhao, 2018 |
| NC_007114.7:25,367,930-25,379,017 | 3 | KPNA2 - karyopherin alpha 2(RAG cohort 1 importin alpha 1) | 1613.8 | 1423.3 | 36808.9 | 31501.7 | 1411.2 | 1057.5 | 1613.8 | Blastula:128-cell (2.25h-2.5h) to Larval:Day 5 (120.0h-144.0h, 3.9mm, 6 teeth) (Zfin.org – ZFIN ID: ZDB-GENE-040718-22) Larval:Day 5 ([bgee.org/](https://bgee.org/) - Gene: kpna2 - ENSDARG00000038066 - Danio rerio (zebrafish)) day 6 and adult ovarian tissue (B.P. Mihalas et al., 2006), S. O’Boyle, R.T. Bree, S. McLoughlin, M. Grealy, L. Byrnes, 2007 | B. Thisse, 2004, Ly-Huynh et al. 2011, B.P. Mihalas et al., 2015, |
| NC_007116.7:54,709,393-54,716,749 | 5 | ccnb1 - Cyclin B1 similar | 658.2 | 627.4 | 81366.3 | 53898.2 | 648.9 | 860.1 | 658.2 | Zygote:1-cell (0.0h-0.75h) to Adult (90d-730d, breeding adult) (zfin.org - Zfin.org – ZFIN ID: ZDB-GENE-000406-10) Larval:Day 5 ([bgee.org/](https://bgee.org/) - Gene: ccnb1 - ENSDARG00000051923 - Danio rerio (zebrafish)), K. Yasuda, T. Kotani, R. Ota, M.Yamashita, 2010, M. Horiea, T. Kotani, 2016 | K. Yasuda, T. Kotani, M. Yamashita 2013; Y. Nagahama and M.Yamashita, 2008; X. Yi, et al. 2019; C. Dingare, et al., 2018; . N. Takei, et al., 2018; T. Kondo, T. Kotani, M. Yamashita, 2001; T. Kondo, T. Yanagawa, N. Yoshida, M. Yamashita, 1997; 7. M. Horie, T. Kotani, 2016; T. Kotani, K. Yasuda, R. Ota, M. Yamashita, 2013; K. Takahashi, T. Kotani, Y. Katsu, M. Yamashita, 2014; A. Knoll-Gellida, et al., 2006 |
| NC_007116.7:44,804,375-44,812,915 | 5 | ctsla - cathepsin La | 10221.3 | 9344.2 | 42760.5 | 37063.7 | 11558.9 | 6157.4 | 10221.3 | Cleavage:16-cell (1.5h-1.75h) to Adult (90d-730d, breeding adult) (zfin.org - ZFIN ID: ZDB-GENE-030131-106) Larval:Day 5 (bgee.org/ - Gene: ctsla - ENSDARG00000007836 - Danio rerio (zebrafish)) | B. Thisse, 2004; A. Tingaud-Sequeira, J. Cerdà, 2007; A. Tingaud-Sequeira, O. Carnevali, J. Cerdà, 2011 |
| NC_007118.7:51,768,240-51,773,562 | 7 | bmp15 | 2.1 | 3.7 | 7428.6 | 5940.5 | 7.2 | 31.1 | 2.1 | Juvenile:Days 30-44 (30d-45d, 10 mm, adult fins/pigment) to Adult (90d-730d, breeding adult) Larval:Day 5 (Zfin.org – ZFIN ID: ZDB-GENE-030131-6115; bgee.org/ - Gene: bmp15 - ENSDARG00000037491 - Danio rerio (zebrafish)). | E. Clelland, et al., 2006; D. B. Dranow, et al., 2016; C. M. Crowder, C. S. Lassiter, D. A. Gorelick, 2018; Y. L. Yan, et al., 2017; S. Hosseini, et al., 2019 |
| NC_007113.7:6,252,741-6,259,344 | 2 | zpb3 - zona pellucida glycoprotein 3 - Fig α transcriotion factor | 1569.3 | 1317.8 | 2724.2 | 2422.8 | 1885.1 | 1571.6 | 1569.3 | Zygote:1-cell (0.0h-0.75h) to Adult (90d-730d, breeding adult) (Zfin.org - ZFIN ID: ZDB-GENE-031121-1) larval day five ([bgee.org/](https://bgee.org/) - Gene: zp3c - ENSDARG00000092919) | A. Jørgensen, J. E. Morthorst, O. Andersen, L. J. Rasmussen, P. Bjerregaard, 2008; D. Schlessinger, A. Forabosco, M. Uda, E. Pelosi, 2011 |
| NC_007115.7:78,068,353-78,068,433 | 4 | Chr4 | 29.6 | 29.6 | 1798.3 | 1808.7 | 0.8 | 14.2 | 29.6 |  | J.L. Anderson, *et al.*, 2012; 50. K. Howe, et al., 2013 |

**Table 6: M genes selected for their role in early sex-determination,** including highest difference of M and F transcriptome data, PCR from this study and additional genes from NGS data. Indicated is the chromosome position, chromosome number, gene name, J, F and M transcriptome expression, the difference in F and M transcriptome data, previous evidence of J and sex-specific expression

| **Chromosome Position** | **Chromosome no.** | **Gene and description** | **juv2** | **juv3** | **f2** | **f1** | **m2** | **m1** | **Diff f-m** | **Juvenile expression** | **Papers Sex determination genetic differences** |
| --- | --- | --- | --- | --- | --- | --- | --- | --- | --- | --- | --- |
| NC_007123.7:1,945,593-1,953,233 | 12 | SOX9a | 1083.6 | 1058.7 | 400.2 | 283.3 | 634.5 | 875.2 | -413.0744826 | Gastrula:Bud (10.0h-10.33h) to Adult (90d-730d, breeding adult) (Zfin.org - ZFIN ID: ZDB-GENE-001103-1), A. Jørgensen, J. E. Morthorst, O. Andersen, L. J. Rasmussen, P. Bjerregaard, 2008 | K. J. Groh, V. J. Nesatyy, H. Segner, R. I. L. Eggen, M. J. F. Suter, 2011; E. F. L. Chiang, et al., 2001; A. T. Major, P. A. F. Whiley, K. L. Loveland, 2011; S. K. Tong, H. J. Hsu, B. C. Chung, 2010; A. Rodríguez-Marí, et al., 2005; V.P. I. Vidal, M.C. Chaboissier, D. G. de Rooij, A. Schedl, 2001; A. Jørgensen, J. E. Morthorst, O. Andersen, L. J. Rasmussen, P. Bjerregaard, 2008; S. Gasca, et al., 2016; N. Klüver, M. Kondo, A. Herpin, H. Mitani, M. Schartl, 2005; G. Yu, et al., 2018; C. M. Crowder, C. S. Lassiter, D. A. Gorelick, 2018; Q. Lin, et al., 2017; D. Sun, et al., 2013; W. Chen, L. Liu, W. Ge, 2017 |
| NC_007127.7:45,042,642-45,071,814 | 16 | gapdhs - glyceraldehyde-3-phosphate dehydrogenase, spermatogenic | 24122.1 | 25901.3 | 13281.5 | 16172.8 | 24304.4 | 26333.8 | -10591.92544 | Blastula:Oblong (3.66h-4.0h) to Adult (90d-730d, breeding adult) (Zfin.org - ZFIN ID: ZDB-GENE-020913-1)), B. Thisse, et al., 2001; J. Liu, C. M. Sun, C. L. Zhang, X. Wang, J. Y. Li, 2013 | D. Paoli, *et al.*, 2016; Y. Fujihara, et al., 2019; 92. G. L. Takei, D. Miyashiro, C. Mukai, M. Okuno, 2014; K. Miki, et al., 2004; J. E. Welch, P. R. Brown, D. A. O. Brien, E. M. Eddy, 1995; M. L. Kuravsky, V. V Aleshin, D. Frishman, 2011; ([bgee.org/](https://bgee.org/) - Gene: gapdhs - ENSDARG00000039914 - Danio rerio (zebrafish)) |
| NC_007117.7:29,215,445-29,229,487 | 6 | atp1b1a - ATPase Na/K transporting, beta 1a polypeptide | 23058.6 | 23810.7 | 9477.7 | 13055.8 | 23207.3 | 24501.6 | -12587.68307 | Gastrula:50%-epiboly (5.25h-5.66h) to Adult (90d-730d, breeding adult) (Zfin.org - ZFIN ID: ZDB-GENE-001127-3), M. Ma, Y. J. Jiang, 2007; L. Wang, et al., 2007; B. Thisse, et al., 2001; L. Abbas, T. T. Whitfield, 2009; J. Hatzold, et al., 2016 | C. J. Martyniuk, E. R. Gerrie, J. T. Popesku, M. Ekker, V. L. Trudeau, 2007 |
| NC_007118.7:25,324,288-25,348,597 | 7 | cyp26b1 cytochrome P450, family 26, subfamily b, polypeptide 1 | 1979.9 | 1291.0 | 1081.0 | 982.1 | 2169.5 | 2221.8 | -1164.099148 | Zygote:1-cell (0.0h-0.75h) to Adult (90d-730d, breeding adult) (Zfin.org - ZFIN ID: ZDB-GENE-030131-2908) | A. Pradhan, P. Olsson, 2015; J. L. Kipp, et al., 2015; K. Kashimada, et al., 2011; J. Bowles, et al., 2016; S. Reijntjes, A. Blentic, E. Gale, M. Maden, 2005; J. Koubova, et al., 2006; A. Rodríguez-Marí, et al., 2013 |

**S7**

**Table 7: Additional genes referred to in main text but not selected for early sex-determination in this study.** Indicated is the chromosome position, chromosome number, gene name, previous evidence of J and sex-specific expression

| **Chromosome poition** | **Chromosome no.** | **Gene name** | **Juvenilles expression** | **Papers Sex determination genetic differences (M or F)** |
| --- | --- | --- | --- | --- |
| NC_007135.7:12,835,571-12,841,666 | 24 | Nanog | Zygote:1-cell (0.0h-0.75h) to Adult (90d-730d, breeding adult) (zfin.org - ZFIN ID: ZDB-GENE-030131-5486) Larval day 5 (uniprot.org - UniProtKB - A5JNG8 (A5JNG8_DANRE)) | **(F)** M. Veil, M. A. Schaechtle, M. Gao, V. Kirner, L. Buryanova, 2018; J. A. Gagnon, K. Obbad, A. F. Schier, 2018; A. V. Sánchez-Sánchez, et al., 2010 |
| NC_007135.7:12,746,168-12,840,136 | 24 | Ipo4 | Larval:Day 5 (uniprot.org - UniProtKB - F1R750 (F1R750_DANRE)) expressed at day 28 (A. T. Major, P. A. F. Whiley, K. L. Loveland, 2011), A. T. Major, P. A. F. Whiley, K. L. Loveland, 2011 | **(F)** F. Xavier, M. Zamorano, 2015 |
| NC_007126.7:7,054,845-7,059,418 | 15 | foxl2a | Larval:Days 14-20 (14d-21d, 6.2mm, 10 teeth) to Adult (90d-730d, breeding adult) (zfin.org - ZFIN ID: ZDB-GENE-060512-241) Larval day 5 (uniprot,org - UniProtKB - F8W2R1 (F8W2R1_DANRE)) | **(F)** Y. J. Yang, Y. Wang, Z. Li, L. Zhou, J. F. Gui, 2017; S. K. Tong, H. J. Hsu, B. C. Chung, 2010; C. M. Crowder, C. S. Lassiter, D. A. Gorelick, 2018; D. Schlessinger, A. Forabosco, M. Uda, E. Pelosi, 2011; |
| NC_007133.7:22,634,007-22,721,110 | 22 | ftz-f1 (also called nr5a2 or ff1a) | Zygote:1-cell (0.0h-0.75h) to Adult (90d-730d, breeding adult) (zfin.org - ZFIN ID: ZDB-GENE-990415-79) Larval:Day 5 (uniprot.org - UniProtKB - O42186 (O42186_DANRE)), S. K. Tong, H. J. Hsu, B. C. Chung, 2010 | **(F)** J. von Hofsten, P. E. Olsson, 2005; C. Chai, W. Chan, 2000; A. Jørgensen, J. E. Morthorst, O. Andersen, L. J. Rasmussen, P. Bjerregaard, 2008; Y. J. Yang, Y. Wang, Z. Li, L. Zhou, J. F. Gui, 2017; J. Koskinen, J. Karlsson, P. Olsson, 2009; J. Von Hofsten, I. Jones, J. Karlsson, P. Olsson, 2001 |
| NC_007125.7:22,456,550-22,464,867 | 14 | gdf9, Growth differentiation factor 9 | Zygote:1-cell (0.0h-0.75h) to Adult (90d-730d, breeding adult) (zfin.org - ZFIN ID: ZDB-GENE-050221-7) | **(F)** E. Clelland, *et al., 2006; W. Chen, L. Liu, W. Ge, 2017* |
| NC_007124.7:28,601,137-28,611,272 | 13 | cyp17a | Zygote:1-cell (0.0h-0.75h) to Adult (90d-730d, breeding adult) (zfin.org - ZFIN ID: ZDB-GENE-040213-2) Larval:Day 5, Y. Yan, et al., 2018 | **(F)** G. Zhai, et al., 2018; G. Yu, et al., 2018; Q. Lin, et al., 2017; H. Xia, et al., 2018; P. P. De Waal, M. C. Leal, N. Hinfray, 2007; Y. L. Yan, et al., 2017; Y. J. Yang, Y. Wang, Z. Li, L. Zhou, J. F. Gui, 2017; Xiaoshan. L, et al., 2016; N. Hinfray, et al., 2011 |
| NM_001115050.1 NC_007119.7:13978555-13985032 | 8 | igf3 | Zygote:1-cell (0.0h-0.75h) to Adult (90d-730d, breeding adult) (zfin.org - ZFIN ID: ZDB-GENE-080611-1) Larval:Day 5 (uniprot.org - UniProtKB - F1QNT1 (F1QNT1_DANRE)), D. S. Wang, et al., 2008; S. K. McMenamin, J. E. N. Minchin, T. N. Gordon, J. F. Rawls, D. M. Parichy, 2013 | **(F)** D. S. Wang, et al., 2008; J. Li, L. Chu, X. Sun, Y. Liu, C. H. K. Cheng, 2015; 21. H. Tang, et al., 2016; H. Xia, et al., 2018; R. H. Nóbrega, et al., 2015; D. Das, et al., 2016; J. Li, Z. Liu, D. Wang, C. H. K. Cheng, 2011 |
| NC_007123.7:24,738,966-24,770,184 | 12 | Gonadotropin receptor fshr | Juvenile:Days 45-89 (45d-90d, 14 mm, 12 teeth) to Adult (90d-730d, breeding adult) (Zfin.org - ZFIN ID: ZDB-GENE-020423-5), A. García-Lopez, et al., 2010 | **(M)** A. García-Lopez, et al., 2010; G. Zhai, et al., 2018; D. Crespo, L. H. C. Assis, T. Furmanek, J. Bogerd, R. W. Schulz, 2016; R. H. Nóbrega, et al., 2015; Morais, et al., 2017 |
| NC_007124.7:47,573,473-47,618,843 | 13 | Gonadotropin receptor lhcgr | Juvenile:Days 45-89 (45d-90d, 14 mm, 12 teeth) to Adult (90d-730d, breeding adult) (Zfin.org - ZFIN ID: ZDB-GENE-040806-3) | **(M)** A. García-Lopez, et al., 2010 |
| NC_007114.7:62,522,542-62,527,667 | 3 | sox9b | Gastrula:Bud (10.0h-10.33h) to Adult (90d-730d, breeding adult) (Zfin.org - ZFIN ID: ZDB-GENE-001103-2) | **(M)** S. K. Tong, H. J. Hsu, B. C. Chung; 2010; N. Klüver, M. Kondo, A. Herpin, H. Mitani, M. Schartl, 2005; W. Chen, L. Liu, W. Ge, 2017 |
| NC_007131.7:31,267,702-31,286,894 | 20 | apobb1 - Apolipoprotein Bb, tandem duplicate 1, transcript variant X1 | Gastrula:50%-epiboly (5.25h-5.66h) to Adult (90d-730d, breeding adult) (Zfin.org - ZFIN ID: ZDB-GENE-030131-9732), J. P. Otis, et al., 2015 | **(M)** B. Thisse, et al., 2001; B. Thisse, C. Thisse, 2004; E. M. Zeituni, et al., 2016; L. Levi, T. Ziv, A. Admon, B. Levavi-Sivan, E. Lubzens, 2012 |
| NC_007114.7:55,089,458-55,113,309 | 3 | hbaa1 - hemoglobin, alpha adult 1 | Zygote:1-cell (0.0h-0.75h) to Adult (90d-730d, breeding adult) (Zfin.org - ZFIN ID: ZDB-GENE-980526-79), B. Thisse, C. Thisse, 2004; A. Roesner, T. Hankeln, T. Burmester, 2006; C. Y. Chu, et al., 2007; T. Nishiyama, et al., 2012; J. J. Ganis, et al., 2012; S. Mercurio, et al., 2015; A. P. Kovina, et al., 2017; Y. Zhong, Q. Ye, C. Chen, M. Wang, H. Wang, 2018 | **(M)** B. C. S. Higano, et al., 1997; J. Tiedke, F. Gerlach, S. A. Mitz, T. Hankeln, T. Burmester, 2011; T. Porseryd, et al., 2017 |
